# Development of Spexin-based Human Galanin Receptor Type II-Specific Agonists with Increased Stability in Serum and Anxiolytic Effect in Mice

**DOI:** 10.1038/srep21453

**Published:** 2016-02-24

**Authors:** Arfaxad Reyes-Alcaraz, Yoo-Na Lee, Gi Hoon Son, Nam Hoon Kim, Dong-Kyu Kim, Seongsik Yun, Dong-Hoon Kim, Jong-Ik Hwang, Jae Young Seong

**Affiliations:** 1 Graduate School of Medicine, Korea University, Seoul 02841, Republic of Korea

## Abstract

The novel neuropeptide spexin (SPX) was discovered to activate galanin receptor 2 (GALR2) and 3 (GALR3) but not galanin receptor 1 (GALR1). Although GALR2 is known to display a function, particularly in anxiety, depression, and appetite regulation, the further determination of its function would benefit from a more stable and selective agonist that acts only at GALR2. In the present study, we developed a GALR2-specific agonist with increased stability in serum. As galanin (GAL) showed a low affinity to GALR3, the residues in SPX were replaced with those in GAL, revealing that particular mutations such as Gln5 → Asn, Met7 → Ala, Lys11 → Phe, and Ala13 → Pro significantly decreased potencies toward GALR3 but not toward GALR2. Quadruple (Qu) mutation of these residues still retained potency to GALR2 but totally abolished the potency to both GALR3 and GALR1. The first amino acid modifications or D-Asn1 substitution significantly increased the stability when they are incubated in 100% fetal bovine serum. Intracerebroventricular administration of the mutant peptide with D-Asn1 and quadruple substitution (dN1-Qu) exhibited an anxiolytic effect in mice. Taken together, the GALR2-specific agonist with increased stability can greatly help delineation of GALR2-mediated functions and be very useful for treatments of anxiety disorder.

The novel neuropeptide spexin (SPX), which is encoded by the *C12ORF39* gene, was originally discovered using bioinformatics tools^1^,^2^. The predicted mature SPX peptide sequence of 14 amino acids flanked by dibasic cleavage sites is evolutionarily conserved across vertebrate species^1^,^2^,^3^,^4^,^5^. SPX expression at the mRNA and/or protein level has been documented in brain regions and peripheral tissues of humans, mice, rats, and goldfish^1^,^2^,^3^,^4^,^5^,^6^,^7^,^8^, suggesting multiple physiological functions of SPX. Recently, SPX was implicated in regulation of feeding behaviors and related metabolic processes. SPX mRNA levels are markedly decreased in the fat of obese humans, and administration of SPX leads to weight loss in diet-induced obese rodents^9^. SPX also suppresses appetite in goldfish^5^. In addition, SPX is likely involved in reproduction, cardiovascular/renal function, and nociception^4^,^10^. The precise roles of SPX in these processes, however, are not well understood due to a lack of information on the SPX receptor. Recently, we demonstrated that SPX is an endogenous ligand that acts at GALR2 and GALR3 but not at GALR1, while GAL activates all three receptor subtypes with relatively low potency and affinity for GALR3^11^.

The SPX and GAL genes likely emerged through a local duplication from a common ancestor gene, and as a result, their mature peptides share several conserved residues, including Trp2, Thr3, Tyr9, Leu10, and Gly12^11^,^12^. Like SPX, GAL is widely expressed in the central nervous system and peripheral tissues^13^,^14^,^15^,^16^. The actions of SPX and GAL in appetite behavior and reproduction, however, appear to oppose each other. For instance, levels of circulating GAL, along with neuropeptide Y and leptin, are significantly higher in obese women^17^, and GAL administration or overexpression in mice results in an increase in food intake^18^,^19^. Thus, GAL appears to be orexigenic, while SPX is anorexic. Administration of galanin-like peptide (GALP), a GAL paralog, stimulates luteinizing hormone (LH) secretion in the rat^20^, while SPX administration attenuates LH secretion in the goldfish^4^. These opposing effects are likely due, at least in part, to GAL receptor subtype-specific signaling pathways. Specifically, GALR1 and GALR3 induce inhibitory G_i_-coupled signaling, while GALR2 triggers stimulatory Gq-coupled signaling^21^.

Considering the cross-reactivity between SPX and GAL, as well as the GAL receptors and the overall complexity of the GAL receptor-mediated signaling pathway, development of more stable and subtype-selective GAL receptor agonists would lead to marked progress in the elucidation of GAL receptor subtype-specific mediated physiological functions. Thus, we focused our studies on the development of SPX sequence-based GALR2-specific agonists, based on the observations that both SPX and GAL have similarly high affinity for the GALR2 and that GAL exhibited a lower affinity and efficacy for the GALR3 than SPX^11^. These findings led to the hypotheses that SPX-based agonists initially prevent the interaction with GALR1 and that replacement of residues in SPX with those of corresponding GAL will decrease the potency of the ligand for GALR3 without altering the affinity for GALR2. As predicted, the present study showed that replacement of SPX-specific residues Gln5, Met7, Lys11, and Ala13 with those of GAL maintained potency to the GALR2 but significantly decreased the potency toward the GALR3. In addition, replacement and modification of Asn^1^ greatly increased the stability of the GALR2-specific agonist in 100% fetal bovine serum (FBS). Considering the relevance of GALR2-mediated pathophysiology, such as anxiety-like behavior^22^, and the putative role of SPX in appetite regulation^9^, a GALR2-specific agonist may prove useful for the treatment of obesity and anxiety disorders.

## Results

### Effects of human SPX1 substitutions on activation of the GAL receptors

**Figure 1:**
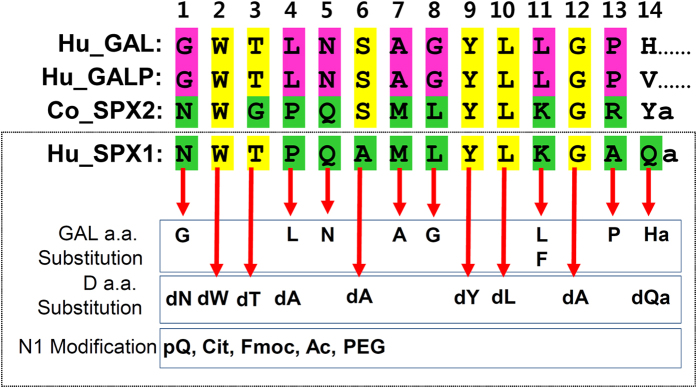
Sequence alignment of human (Hu) GAL, GALP, SPX1, and coelacanth (Co) SPX2. Conserved residues in the peptides are indicated in yellow. SPX-specific residues are in green, while GAL-specific residues are highlighted in violet. The residues in human SPX1 were changed as indicated.

To develop a specific and long-acting GALR2-specific agonist, we compared the amino acid sequences of SPX1, SPX2, GAL, and GALP. These peptides share the residues Trp2, Tyr9, Leu10 and Gly12, while other residues are specific to GAL or SPX or variable across vertebrate species (Fig. 1). The SPX-specific residues in SPX1 were replaced with the corresponding GAL-specific residues. D-amino acids were substituted for residues common to SPX and GAL in SPX1.

**Figure 2:**
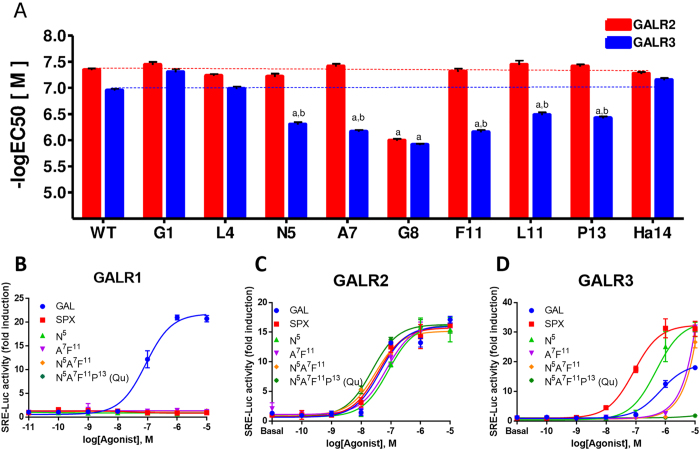
Differential effects of GAL-specific residue substitution in SPX on potencies toward GALR1, GALR2, and GALR3. The SRE-luc reporter vector and plasmid containing human GALR1, GALR2, or GALR3 were transfected into HEK293 cells that stably expressed Gq_i_ protein. Cells were then treated with increasing concentrations of mutant peptides, and the receptor activities were determined using the SRE-luc reporter system. (**A**) Potencies of mutant peptides toward GALR2 (red bars) and GALR3 (blue bars) are expressed as −log[EC_50_] values. The horizontal dashed lines represent potency of WT SPX toward receptors. (**B**–**D**) Dose-response activity fold induction by peptides with multiple substitutions on GALR1 (**B**), GALR2 (**C**), and GALR3 (**D**).WT SPX and GAL were used as control peptides. a, *P* < 0.05 vs. WT SPX; b, *P* < 0.05 vs. activity with GALR2.

The generated mutant SPX peptides were incubated with cells expressing GALR2 and GALR3. Because each GAL receptor subtype is coupled to a different G protein (G_i_ for GALR1 and GALR3; G_q/11_ for GALR2^21^), we employed the HEK293-G_qi_ stable cell line for these experiments^11^. The activities of the receptors were traced using the serum-response element-driven luciferase (SRE-luc) assay system. Of these, the Gly8 substitution resulted in decrease in the potency toward both receptors, while the Gly1, Leu4, and His14 substitutions retained potencies to both receptors that are similar to WT SPX. Interestingly, Asn5-, Ala7-, Leu11-, Phe11-, and Pro13-substituted mutants showed a selective decrease in the potency for the GALR3 without a concomitant effect on the activation profile for the GALR2. This result suggests that SPX residues Gln5, Met7, Lys11, and Ala13 are responsible for the selectivity for the GALR3, while substitutions of these residues are acceptable for activation of the GALR2 (Fig. 2A and Table 1).

In order to further support this notion, we generated SPX peptides with combined substitutions of Asn5, Ala7, Phe11, and/or Pro13. Double (A7F11) and triple (N5A7F11) substitutions greatly decreased the potency toward GALR3 (Fig. 2B–D). These mutant peptides exerted full activation of the GALR3 only at a high concentration (10 μM). The quadruple substitution (N5A7F11P13, Qu) completely abolished activation of the GALR3 but retained the potency to the GALR2 in the manner similar to that of WT SPX. More importantly, introduction of any of the GAL-specific residues did not induce activation of the GALR1 (Fig. 2B–D).

**Figure 3:**
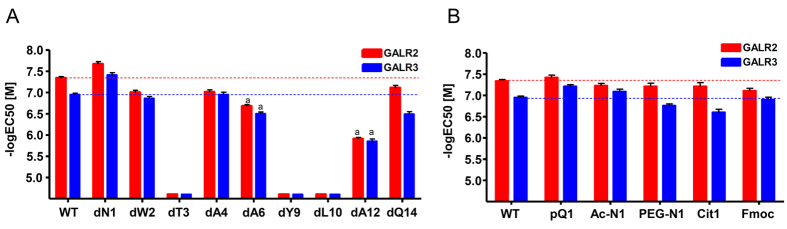
Effect of single D-amino acid substitution (**A**) and Asn1 replacement/modification (**B**) on the potency toward GALR2 and GALR3. The SRE-luc reporter vector and plasmid containing human GALR2 or GALR3 were transfected into HEK293 cells that stably expressed Gq_i_ protein. Cells were then treated with increasing concentrations of mutant peptides, and the receptor activities were determined using the SRE-luc reporter system. Potencies of mutant peptides toward the GALR2 (red bars) and GALR3 (blue bars) by the mutant peptides are expressed as −log[EC_50_] values. The horizontal dashed lines represent potency of WT SPX toward receptors. a, *P* < 0.05 vs. WT SPX

The purpose of D-amino acid substitution is to identify residues that are responsible for receptor activation and to determine residues that are tolerant of D-amino acid substitution, which functions to protect the peptide from attacks by a large variety of proteases present in serum. Following replacement of the SPX residues with their D-amino acids, the mutant peptides were incubated with cells expressing GALR2 and GALR3. Of the mutant SPX peptides, dT3, dY9, and dL10 mutants exhibited a drastic loss of activity toward both GALR2 and GALR3 (Fig. 3A), suggesting that the residues Thr3, Tyr9, and Leu10 may play a critical role in the activation of both receptors. The moderate loss of activity toward both receptors was observed for dA6, dA12, and dQ14 mutants. The dAla4 and dTrp2 substitutions only slightly affected the activation of both receptors, suggesting that the orientation of amino acids Trp2 and Ala4 is not crucial for activation of either of the receptors.

Notably, the dAsn1 amino acid substitution slightly increased potency toward both receptors (Fig. 3A and Table 2). This observation spurred additional modifications of Asn1 in an attempt to obtain agonists that are more potent than WT SPX and stable in the presence of serum proteases. Thus, Asn1 was replaced with pyroglutamate (pQ), citrulline (Cit), or functionalized with a fluorenylmethoxycarbonyl (Fmoc) moiety. Alternatively, its N-terminus was acetylated (Ac-N1) or polyethylene glycosylated (PEG-N1) (Fig. 1). Like the dAsn1 substitution, these modifications or substitutions did not significantly alter potency toward both the GALR2 and GALR3 (Fig. 3B and Table 2). These data suggest that Asn1 modifications do not significantly decrease the peptide activity and that these modifications may be important for improving the stability of the agonists in serum.

### Serum Stability of SPX and Qu analogs

**Figure 4:**
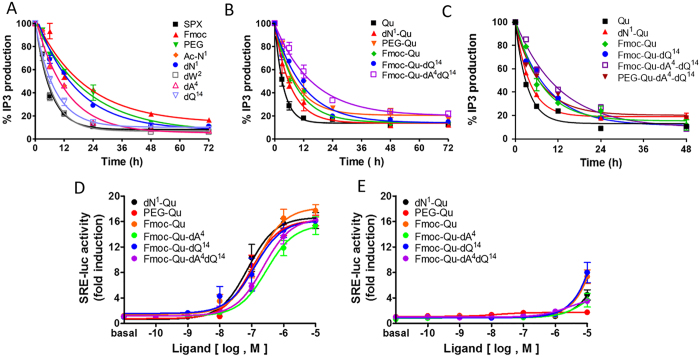
Stability of SPX mutants in the presence of FBS (**A**) Qu analogs in FBS (**B**) and human serum (**C**). Potency of Qu mutants toward GALR2 (**D**) and GALR3 (**E**). For stability of SPX mutants, peptides (1 μM) were incubated in serum for 0, 3, 6, 12, 24, 48, and 72 hrs. The remaining activities of the peptides were determined by measuring IP3 production by HEK293 cells that express GALR2 after the indicated times. IP3 production was expressed as percent change, where 100% indicates IP3 production by WT SPX incubated in FBS or human serum for 0 h. For potency of Qu mutants toward GALR2 and GALR3, the SRE-luc reporter vector and plasmid containing human GALR2 or GALR3 were transfected into HEK293 cells that stably expressed Gq_i_ protein. Cells were then treated with increasing concentrations of mutant peptides, and the receptor activities were determined using the SRE-luc reporter system.

To investigate the effects of the modifications on proteolytic susceptibility, the functional activity of peptides that were incubated in 100% serum at 37 °C for 0, 3, 6, 12, 24, 48, and 72 h was determined via inositol 1,4,5-triphosphate (IP3) production in GALR2-expressing cells (Fig. 4A–C and Table 3). The activity of WT SPX rapidly decreased with time, and a 12-h incubation in FBS abolished 80% of the initial activity. The peptides with N-terminus modifications as well as the Ala4 substitution showed better stability with decreased IP3 production values compared to WT SPX (Fig. 4A). This result suggests that protection of the N-terminal of SPX with PEG or hydrophobic molecules such as Fmoc results in a significant increase in the stability in serum. In addition, dAla4 and dGln14 substitutions contribute to longer longevity of the peptide in serum. In an additional experiment, we examined the degradation rates of the Qu mutant, as well as the Qu mutant with an N-terminal modification and/or dAla4 and dGln14 substitutions. The Qu mutant exhibited a faster decay rate than WT SPX. The N-terminal modification PEG, Fmoc and the dAla4 and dGln14 double substitution of Qu greatly increased the longevity of the peptide in FBS and human serum, respectively (Fig. 4B,C).

### Potency of Qu mutants toward GALR2 and GALR3

We then examined the activities of the Qu analogs toward the GALR2 and GALR3. Compared to the potency of Qu peptide toward the GALR2, all Qu mutants exhibited slightly lower activities with the GALR2, although these differences were not statistically significant (Fig. 4C). The partial activity of the Qu mutants with the GALR3 was observed only at a concentration of 10 μM (Fig. 4D). Thus, all these Qu mutants are highly specific to the GALR2 and have higher longevity in serum compared to WT SPX and Qu.

### GALR2-specific agonist exerts anxiolytic effect in a rodent model

**Figure 5:**
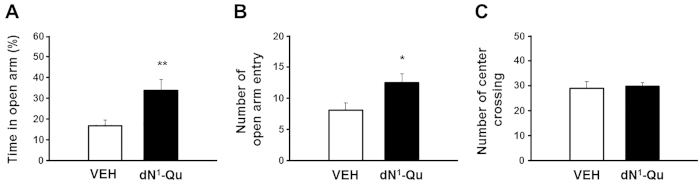
Administration of the GALR2 agonist dN1-Qu displays anxiolytic activity in animal models. Graphs show the % time in open arm (**A**), number of open arm entry (**B**) and center crossing (**C**). Data are presented as mean ± SEM (n = 9 for VEH and n = 8 for dN1, *p < 0.05 and **p < 0.01 versus VEH and dN1).

To examine effect of GALR2 specific agonist *in vivo*, Qu mutant peptide with dAsn^1^ substitution (dN1-Qu) was intracerebroventricularly administered into the lateral ventricle of mice. Anxiolytic effect of dN1-Qu was examined in the elevated plus-maze. Duration (Fig. 5A) and frequency (Fig. 5B) of open arm entries were significantly increased in the dN1-Qu-injected mice compared to those of vehicle-injected mice. The number of center crossing did not differ between the vehicle- and dN1-Qu-treated group (Fig. 5C), suggesting that the anxiolytic behavior of dN1-Qu-treated mice is not due to hyperactivity. These results demonstrate that the GALR2-specific agonist decreases anxiety-like behavior in rodent model.

## Discussion

The actions of SPX and GAL are mediated by three GAL receptor subtypes. SPX activates GALR2 and GALR3 with high potency and high affinity, while GAL interacts with all three subtypes, albeit with relatively low potency and efficacy for GALR3^11^. The detailed mechanism of SPX and GAL activity on each GAL receptor subtype is largely unclear. Thus, studies on each receptor function would benefit from the development of more stable and highly selective agonists. Thus, the development of subtype-specific agonists has been the focus of research for both delineating the mechanisms for subtype-specific pathophysiological functions and treating subtype-specific disorders, such as anti-nociception and seizure delay^21^,^23^,^24^. A GAL fragment, GAL (2–11), was first suggested as a GALR2 selective agonist^25^, but further studies unfortunately revealed that this fragment has similar affinity for the GALR3^26^. Several GALR2-specific agonists, which were generated by modification at the N-terminus and/or C-terminus of GAL, have been reported over the years^21^. Of these, M1145^27^ and M1153^28^ were found to exhibit GALR2 selectivity with 50–100-fold binding preference for GALR2 compared to GALR1 and GALR3; however, at high concentrations, these agonists retain substantial affinity for GALR1 and GALR3^21^.

The GALR2-specific agonists developed in this study do not activate GALR1 and GALR3 even at a concentration of 10 μM. In addition, these agonists exhibited potency similar to or better than wild-type SPX and GAL. Our approach for developing GALR2-specific agonists differs from previous approaches that were largely based on the GAL sequence. Our GALR2-specific agonists are based on the SPX sequence, whereby possible interaction with the GALR1 is eliminated. Lower potency and affinity of GAL toward GALR3 supported the notion that substitution of GAL-specific residues for corresponding residues in SPX could decrease the interaction with the GALR3. Indeed, we found that substitutions (Gln5 → Asn, Met7 → Ala, Lys11 → Phe, and Ala13 → Pro) greatly decreased the potency to GALR3. The quadruple mutation completely abolished all activation of the GALR3, while activation of the GALR2 remained as potent as that by wild-type SPX.

In addition to this GALR2 specificity, we also investigated the stability of the agonists in serum, which contains a large variety of proteases^29^,^30^, as this is a particularly important issue for future clinical applications. Incubation of wild-type SPX in 100% FBS resulted in a drastic decrease of its activity in a time-dependent manner; however, the modifications of the N-terminal amino acid Asn1 by substitutions of dAsn1, addition of Fmoc, acetylation or polyethylene glycosylation of Asn1, greatly increased the stability of the peptides up to four times compared to wild-type SPX. As the N-terminal modification or substitutions of D-amino acid for N- and C-terminal residues increased stability of the peptides in serum, the cleavage of the terminal peptide bonds by serum proteases^31^ is likely one of the main reasons accounting for rapid degradation of the peptide. In addition, d-Ala4 substitution but not d-Trp2 substitution significantly increased the stability of the peptide, indicating that the residue at position 4 seems to be a possible cleavage site of the peptide by serum proteases. On the other hand, polymer conjugation is an efficient and relatively easy way of modifying the pharmacokinetic properties of peptides or small-molecule drugs. For instance, a PEG polymer conjugated to the peptide becomes tightly associated with two or three water molecules, which acts like a shield to protect the N-terminal of the peptide from enzymatic degradation^32^. Thus, combination of the quadruple mutation with N-terminal modifications and/or D-amino acid substitutions provides GALR2-specific agonists with increased longevity in serum, and these agonists may be useful clinically for GALR2-mediated pathophysiological conditions, effectively avoiding GALR1 or GALR3-mediated interference.

The functions of GALR2 have been postulated based on observations using *GALR2* knockout (KO) or knockdown mice and animal models treated with GALR2-specific agonists and antagonists. In an initial study using GALR2 KO mice, no unusual phenotypes with respect to basic motor and sensory function, feeding behavior, reproduction, mood, learning and memory, and seizure susceptibility were observed compared with wild-type littermates^33^. Later then, two independent groups demonstrated anxiety- and depression-related behaviors in GALR2 KO mutants^22^,^34^. This phenotype was similar to that observed in GALR1 KO mice^35^; however, this GALR2-mediated effect is likely the opposite of the GALR3 effect, as GALR3-specific antagonists decrease anxiety and induce depression-like behavior in rats^36^. In this work we demonstrate that GALR2-specific agonist display an acute anxiolytic behavioral profile in the elevated plus-maze. As this agonist does not act at GALR3, the neutralization effect through GALR3 can be excluded.

In addition to anxiety- and depression-related behaviors, GALR2 deficiency resulted in developmental loss of dorsal root ganglion neurons, suggesting a possible role in pain behavior^37^. Indeed, microinjection of a GALR2-specific agonist into the spinal cord induces allodynic effects^25^. In addition, GAL through the GALR1, but not the GALR2, negatively regulates the action of morphine that leads to opiate dependence and withdrawal^38^. Involvement of GALR2 in the mesolimbic reward system has been reported; GAL decreases the amplitude of excitatory postsynaptic potential in dorsal striatum and nucleus accumbens, and this effect is absent in GALR2 KO mice^39^. In the central amygdala, GAL, through binding of the GALR2, decreases the amplitudes of pharmacologically isolated GABAergic inhibitory postsynaptic potentials^40^. GALR2 also plays a protective role in hippocampal cultures. Activation of GALR2 protects the hippocampus from neuronal damage through stimulation of the phosphorylation of the serine/threonine kinase Akt and extracellular signal-regulated kinase in wild-type hippocampal cultures. This effect is markedly attenuated in GALR2 KO mice^41^. Similarly, GALR1 deletion exacerbates hippocampal neuronal loss after systemic kainate administration in mice^42^. Activation of dentate gyrus GALR2 by a specific agonist causes transient attenuation of long-term potentiation^43^, while *GALR2* knockdown in the dentate gyrus causes serious seizures in rats^44^. Overall, GALR2-mediated signaling pathways function in opposition, in concert, or independently of GALR1 and GALR3- mediated signaling, due to the complex nature of the GAL receptor system^21^,^45^. Nevertheless, these observations suggest the possible involvement of GALR2s in learning and memory, seizure, pain, anxiety, and mood disorders.

GAL and SPX also play a role in reproduction and feeding behaviors, as GAL receptors are expressed in the arcuate, dorsomedial, and ventromedial nuclei of the hypothalamus, where regulation of appetite takes place^46^,^47^,^48^; however, the receptors that are involved in these processes are unknown. GAL exerts a stimulatory effect on the secretion of gonadotropin-releasing hormone and LH in mammals^20^,^49^,^50^. SPX, however, suppresses LH secretion from the gonadotrope of sexually mature female goldfish^4^. The involvement of GAL in feeding behavior has also been demonstrated^49^,^51^. Centrally administered GALP may eventually induce a reduction of food intake^51^. Both central and peripheral injections of SPX1 into goldfish result in the prompt inhibition of food intake and alteration of feeding behavior^5^.

Our mutation study takes into account the evolutionary fates of duplicated receptors and peptide ligand genes. The whole-genome duplication produced GALR2 and GALR3 from a GALR2/3 progenitor. This event was preceded by a local duplication that generated GALR2/3 and GALR1 progenitors. Likewise, GAL and SPX progenitors originated from a common ancestor via a local duplication, and following whole-genome duplication, this progenitor produced the GAL family (GAL and GALP) and SPX family (SPX1 and SPX2)^11^,^12^. Duplication of a gene provides an opportunity for one of the daughter genes to undergo rapid mutation of its sequence, while the other gene undergoes relatively slow mutation, allowing one daughter gene to maintain the original functions while the other acquires additional functions, the so-called “neofunctionalization” or “subfunctionalization”^52^,^53^,^54^,^55^,^56^. Mutation of the receptor accompanies the mutation of the peptide genes in order to retain the high-affinity interaction between the ligand and receptor^56^,^57^,^58^. Based on our observations, Gln5, Met7, Lys11, and Ala13 of SPX are critical for high-affinity interactions with GALR3, but replacement of these residues with those of the corresponding residues of GAL does not alter the peptide affinity to GALR2. Therefore, changes of these residues to either those from GAL or SPX are major determinants for selectivity toward each GAL receptor; during the divergence of the GAL/SPX and GALR1/2/3 system, the GALR2 appears to have become an intermediate form that responds to both SPX and GAL with high affinity, whereas GALR1 and GALR3 acquired significant preference to GAL and SPX, respectively. Interestingly, the GALR2 mainly couples to the stimulatory G_q/11_ protein, while GALR1 and GALR3 interact with the inhibitory G_i_ protein. Thus, the evolutionary relationship between two ligand families and three receptors with different signaling cascades in concert with the differential and/or similar expression pattern of the ligand and receptor genes may explain the varied functions of GALR2 (sharing, opposing, or independent) compared to those of GALR1 and GALR3.

In summary, the complex nature of the GAL receptor system and the absence of specific agonists for each GAL receptor largely hamper our basic understanding of the functions of each GAL receptor subtype. The present study describes SPX-based GALR2-specific agonists that will serve as an important tool for discriminating the function of the GALR2 from those of the GALR1 and GALR3. The additional modification of Asn1 of the GALR2-specific agonist significantly increased the stability of the peptides in serum, allowing possible clinical applications for GALR2-mediated disorders or diseases, such as anxiety, depression, and obesity, as these molecules are known to play important roles in these processes.

## Methods

### Chemicals and peptides

All chemicals were obtained from Sigma-Aldrich (St. Louis, MO, USA) unless otherwise stated. Restriction enzymes were obtained from New England BioLabs (Ipswich, MA, USA). Human GAL, SPX, and all modified SPX peptides were synthesized by AnyGen (Gwangju, Korea). The purity of the synthesized peptides was greater than 98% as determined by high-performance liquid chromatography analysis. All peptides were dissolved in DMSO and then diluted in media to the desired working concentrations.

### Plasmid constructs

The pcDNA3.1 expression vector was purchased from Invitrogen Corp. (San Diego, CA, USA). The SRE-luc vector was purchased from Stratagene (La Jolla, CA, USA). The cDNAs for human GALR1, GALR2 and GALR3 were obtained from BRN SCIENCE Inc. (Seoul, Korea). The cDNA genes were inserted into the *EcoR*I and *Xho*I sites of pcDNA3.1. The identity of each gene was verified by sequencing.

### Cell transfection and luciferase assay

HEK293 cells stably expressing Gα_qi_, which mediates Gα_i_-coupled receptor activation by stimulating Gα_q_-dependent signaling pathways^59^, were maintained in Dulbecco’s modified Eagle’s medium (DMEM) supplemented with 10% FBS, 100 U/ml penicillin G, and 100 μg/ml streptomycin (Invitrogen). Cells were seeded in 48-well plates at a density of 2 × 10^4^ cells per well, one day before transfection. A mixture containing 75 ng of the SRE-luc reporter construct, 75 ng of expression plasmid, and 2 μl of Effectene reagent (Qiagen, Chatsworth, CA, USA) was prepared and added to each well according to the manufacturer’s instructions. Cells were then maintained in serum-free DMEM for 16–18 h before treatment with the ligands. Approximately 48 h after transfection, cells were treated with the respective ligands for 6 h. Cells were lysed by the addition of 100 μl lysis buffer. The luciferase activity in 50 μl of cell extract was determined by a luciferase assay system according to the standard protocol for the Synergy 2 Multi-Mode Microplate Reader (BioTek, Winooski, VT, USA).

### Analysis of peptide stability in serum by IP3 production

To evaluate the stability of SPX and its analogs in FBS or human serum, peptides were incubated at an initial concentration of 1 μM in 100% serum at 37 °C for 0, 3, 6, 12, 24, 48, and 72 hours. Residual peptide activity was then assessed by measuring IP3 in HEK293 cells expressing GALR2. For the IP3 assay, cells were seeded in 12-well plates at a density of 2.5 × 10^5^ cells per well, and then the next day, cells were transiently transfected with 1 μg/well of plasmid containing GALR2 using the Lipofectamine 2000 (Invitrogen) transfection reagent. The day following transfection, cells were incubated in M199 media containing 1% FBS, 1% L-glutamine, 1% penicillin/streptomycin, and 1 μCi/well of myo-^3^H inositol for 20 h. After a 30-min incubation in buffer A (140 mM NaCl, 20 mM Hepes, 4 mM KCl, 8 mM D-glucose, 1 mM MgCl_2_, 1 mM CaCl_2_, 1 mg/ml free fatty acid bovine serum albumin (BSA), and 10 mM LiCl at pH 7.2), cells were treated with the agonist for 40 min at 37 °C. Media was removed, and the reactions were terminated by addition of 1 ml of 10 mM cold formic acid to each well. The plates were stored at 4 °C for 30 min, and the extracts were then transferred into 6-ml plastic tubes containing 500 μl AG1–8X anion exchange columns (BIO-RAD). The tubes were gently mixed with a vortexer, and the supernatants were removed by aspiration. Two washes with 1 ml of distilled water were performed followed by two additional washes with 60 mM ammonium formate/5mM sodium tetraborate. Tritiated inositol was separated from the column by elution with 1 ml of 1 M ammonium formate/0.1 M formic acid, and 800 μl from the elution were taken from each tube and transferred into 6-ml scintillation vials. Then, 2 ml of scintillation cocktail solution (Ultima Gold^TM^, Perkin Elmer, Waltham MA, USA) was added to each sample. The radioactivity of [^3^H]-myo-inositol samples was measured using a TRI carb 3100TR liquid scintillation analyzer (Packard).

### Animals

C57BL/6J strain male mice (9–12 weeks of age) were acquired from Orientbio (Seongnam, Korea). Mice were housed under a normal 12:12 light-dark cycle (lights onset at 8:00 am), with food and water available *ad libitum*. All animal experiments were approved by, and performed in accordance with the guidelines for the institutional animal care and use committee of Korea University (KUIACUC-2015-83).

### Drug administration

For intracerebroventricular injection, mice were anesthetized with sodium pentobarbital (50 mg/kg, intraperitoneal), mounted on a stereotaxic apparatus (stoelting), and implanted with a 26-guage stainless steel cannula into the right side of the lateral ventricle (AP −0.55 mm, ML +1.1 mm, DV −2.05 mm). A 32-guage dummy cannula was inserted into each guide cannula to prevent clogging. Two jewelry screws were implanted into the skull as anchors, and the whole assembly was affixed to the skull with dental cement. Mice were recovered at least 2 weeks before experimentation. dN1-Qu dissolved in DMSO to a concentration of 1.6 μg/μl and then administered into the lateral ventricle (0.8 μg/mouse) 3 hr before experiments using a 33-guage injector cannula (PlasticOne) attached to a 10-μl Hamilton syringe at a rate of 0.2 μl/min.

### Elevated Plus-Maze test

Elevated plus maze (EPM) was performed as previously described^60^. The maze consisted of two open (5 × 30 cm) and two enclosed (5 × 30 cm) arms with 20 cm high walls. The apparatus was elevated 50 cm above the ground. Mice were individually placed in the center of the apparatus facing an open arm and allowed to explore for 10 min. Frequency and duration of arm entries were recorded. An entry was defined as the movement of all four paws into an arm. The percentage of time spent in open arms was scored.

### Statistical analysis

For *in vitro* studies data analysis was performed by nonlinear regression with a sigmoidal dose-response. The agonist concentrations that induced half-maximal stimulation (EC_50_) or half-maximal inhibition of binding (IC_50_) were calculated with GraphPad PRISM4 software (GraphPad Software Inc., San Diego, CA, USA). For animal experiments the data are presented as means ± SEM. Statistical differences between individual groups were evaluated using Student’s *t* test. All data are presented as mean ± S.E. of at least three independent experiments and a P value of P < 0.05 was considered statistically significant.

## Additional Information

**How to cite this article**: Reyes-Alcaraz, A. *et al.* Development of Spexin-based Human Galanin Receptor Type II-Specific Agonists with Increased Stability in Serum and Anxiolytic Effect in Mice. *Sci. Rep.* **6**, 21453; doi: 10.1038/srep21453 (2016).

## Figures and Tables

**Table 1:** EC_50_ and E_max_ values of GAL-specific residue substitutions in SPX.

Substitution	GALR2		GALR3	
	EC_50_(nM)	E_max_(Fold induction)	EC_50_(nM)	E_max_(Fold induction)
WT	45.7 ± 5.90	16.28	112.20 ± 14.48	30.77
G1	36.31 ± 7.47	15.99	93.32 ± 20.88	31.64
L4	58.88 ± 7.59	16.89	104.71 ± 17.61	30.52
N5	61.66 ± 12.68	15.79	501.187 ± 184.32^a^^,^^b^	34.13
A7	38.90 ± 7.28	17.14	467.74 ± 69.63^a^^,^^b^	36.69
G8	1023.29 ± 132.04^a^	17.28	1230.27 ± 108.25^a^	35.27
F11	48.98 ± 10.07	17.36	707.95 ± 119.10^a^^,^^b^	34.29
L11	36.31 ± 10.01	16.88	331.131 ± 61.98^a^^,^^b^	40.04
P13	38.90 ± 5.80	15.99	380.19 ± 49.06^a^^,^^b^	33.63
Ha14	53.70 ± 6.92	16.29	70.79 ± 10.54	32.59
A7F11	144.54 ± 27.05	16.36	>10,000	NM
N5A7F11	107.15 ± 20.06	15.62	>10,000	NM
N5A7F11H14	54.54 ± 10.77	16.95	3019.95 ± 978.21	40.58
N5A7F11P13(Qu)	54.95 ± 9.24	16.26	NM	NM

^a^P < 0.05 *vs.* WT SPX

^b^P < 0.05 *vs.* potency toward GALR2. NM, Not measurable.

**Table 2:** EC_50_ values of D-amino acid substituted and Asn1 substituted/modified peptides for GALR2 and GALR3.

Substitution/Modification	GALR2		GALR3	
	EC_50_(nM)	E_max_(Fold induction)	EC_50_(nM)	E_max_(Fold induction)
dN1	21.38 ± 5.54	15.22	38.90 ± 8.00	36.80
dW2	100.00 ± 18.71	16.94	138.04 ± 23.22	33.01
dT3	NM	NM	NM	NM
dA4	97.72 ± 18.29	16.5	112.20 ± 23.07	27.51
dA6	208.93 ± 26.96^a^	17.14	316.23 ± 47.07^a^	31.76
dY9	NM	NM	NM	NM
dL10	NM	NM	NM	NM
dA12	1230.26 ± 158.75^a^	17.19	1412.54 ± 210.27^a^	37.2
dQa14	77.62 ± 15.97	15.89	323.59 ± 66.55^a^	33.88
pQ1	35.01 ± 7.82	15.85	61.66 ± 10.37	33.08
Ac-N1	58.88 ± 11.02	16.73	81.28 ± 16.71	31.01
PEG-N1	61.65 ± 16.99	14.99	173.78 ± 25.87	31.39
Cit1	61.66 ± 19.00	16.26	251.19 ± 64.98	30.40
Fmoc	77.62 ± 15.97	15.24	125.89 ± 25.89	31.77

^a^P < 0.05 *vs.* WT SPX, NM, Not Measurable.

**Table 3:** Half-life activity of SPX and SPX mutant peptides in serum.

Peptide	Half-life activity(h) in FBS	Peptide	Half-life activity(h) in FBS	Half-life activity(h) in Human Serum
SPX	4.1 ± 0.5	N5A7F11P13 (Qu)	2.6 ± 0.2	2.0 ± 0.2
Fmoc-SPX	14.2 ± 0.2^a^	dN1-Qu	4.2 ± 0.1^a^	2.9 ± 0.1^a^
PEG-SPX	12.5 ± 0.5^a^	PEG-Qu	5.7 ± 0.6^a^	ND
Ac-N1	8.7 ± 0.6^a^	Fmoc-Qu	6.1 ± 0.2^a^	4.9 ± 0.1^a^
dN1	10.3 ± 0.3^a^	Fmoc-Qu-dQ14	8.4 ± 0.3^a^	6.3 ± 0.1^a^
dA4	8.7 ± 0.4^a^	Fmoc-Qu-dA4-dQ14	11.8 ± 0.2^a^	8.8 ± 0.2^a^
dW2	4.5 ± 0.6	PEG-Qu-dA4-dQ14	ND	6.0 ± 0.2 a
dQ14	5.6 ± 0.2^a^			

^a^P < 0.05 *vs.* WT SPX or Qu; ND, Not determined.
